# Fusarium Head Blight Resistance QTL in the Spring Wheat Cross Kenyon/86ISMN 2137

**DOI:** 10.3389/fmicb.2016.01542

**Published:** 2016-10-13

**Authors:** Curt A. McCartney, Anita L. Brûlé-Babel, George Fedak, Richard A. Martin, Brent D. McCallum, Jeannie Gilbert, Colin W. Hiebert, Curtis J. Pozniak

**Affiliations:** ^1^Agriculture and Agri-Food Canada, Morden Research and Development CentreMorden, MB, Canada; ^2^Department of Plant Science, University of ManitobaWinnipeg, MB, Canada; ^3^Agriculture and Agri-Food Canada, Ottawa Research and Development CentreOttawa, ON, Canada; ^4^Agriculture and Agri-Food Canada, Charlottetown Research and Development CentreCharlottetown, PEI, Canada; ^5^Crop Development Centre, University of SaskatchewanSaskatoon, SK, Canada

**Keywords:** Fusarium head blight, *Fusarium graminearum*, wheat, *Triticum aestivum* L., SNP, QTL, linkage

## Abstract

Fusarium head blight (FHB), caused by *Fusarium graminearum*, is a very important disease of wheat globally. Damage caused by *F. graminearum* includes reduced grain yield, reduced grain functional quality, and results in the presence of the trichothecene mycotoxin deoxynivalenol in Fusarium-damaged kernels. The development of FHB resistant wheat cultivars is an important component of integrated management. The objective of this study was to identify QTL for FHB resistance in a recombinant inbred line (RIL) population of the spring wheat cross Kenyon/86ISMN 2137. Kenyon is a Canadian spring wheat, while 86ISMN 2137 is an unrelated spring wheat. The RIL population was evaluated for FHB resistance in six FHB nurseries. Nine additive effect QTL for FHB resistance were identified, six from Kenyon and three from 86ISMN 2137. *Rht8* and *Ppd-D1a* co-located with two FHB resistance QTL on chromosome arm 2DS. A major QTL for FHB resistance from Kenyon (*QFhb.crc-7D*) was identified on chromosome 7D. The QTL *QFhb.crc-2D.4* from Kenyon mapped to the same region as a FHB resistance QTL from Wuhan-1 on chromosome arm 2DL. This result was unexpected since Kenyon does not share common ancestry with Wuhan-1. Other FHB resistance QTL on chromosomes 4A, 4D, and 5B also mapped to known locations of FHB resistance. Four digenic epistatic interactions were detected for FHB resistance, which involved eight QTL. None of these QTL were significant based upon additive effect QTL analysis. This study provides insight into the genetic basis of native FHB resistance in Canadian spring wheat.

## Introduction

Fusarium head blight (FHB), primarily caused by *Fusarium graminearum* Schwabe (teleomorph: *Gibberella zeae* (Schwein.) Petch), is one of the most serious diseases of wheat. FHB lowers grain yield, grain quality, and contaminates grain with the trichothecene mycotoxin deoxynivalenol, and its acetylated derivatives 3-ADON and 15-ADON (Ward et al., 2008). FHB damage reduces functional performance of wheat for bread and noodle production (Dexter et al., 1996; Hatcher et al., 2003) and durum wheat (Dexter et al., 1997). Trichothecenes are a virulence factor for the pathogen and have multiple inhibitory effects on eukaryote cells, which are harmful to the plant host and any humans and animals consuming contaminated grain (Proctor et al., 1995; Rocha et al., 2005). FHB has been a problem in eastern Canadian wheat since the 1980s, and only became a significant problem in western Canada in 1993, particularly in the province of Manitoba (Gilbert and Tekauz, 2000). In 2014, FHB caused substantial damage in the province of Saskatchewan (https://www.grainscanada.gc.ca/str-rst/fusarium/data/frequency-en.htm, accessed 25 April 2016), which had been largely unaffected by FHB until 2014.

Host FHB resistance is an important control measure and wheat breeding objective. The genetic basis of FHB resistance in Asian spring wheats has been the focus of many genetic studies. Buerstmayr et al. (2009) reported 52 QTL for FHB resistance in a comprehensive review of published research. Similarly, (Liu et al., 2009) and (Löffler et al., 2009) performed meta-QTL analyses and identified 43 QTL clusters and 19 important QTL, respectively. A few FHB resistance QTL have been studied in isolation from other FHB resistance QTL, which enabled the resistance controlled by these QTL to be treated as a qualitative trait and mapped as discrete loci, *Fhb1* (Cuthbert et al., 2006; Liu et al., 2008) and *Fhb2* (Cuthbert et al., 2007).

The genetic basis of FHB resistance in Canadian spring wheat germplasm is not well understood. Native FHB resistance is a topic that has gained interest as wheat breeders have struggled to make progress in improving FHB resistance using exotic resistance sources. There is a strong need for wheat breeders to understand the basis of FHB resistance already present in their programs so that introgression of FHB resistance QTL from exotic germplasm is targeted. In western Canada, a number of hard red spring wheat varieties have been identified that possess an intermediate level of FHB resistance relative to more highly susceptible wheat varieties, but do not have Asian sources of FHB resistance in their pedigrees. Such Canadian varieties include AC Barrie, CDC Bounty, AC Cadillac, AC Cora, Journey, Kane, Katepwa, McKenzie, Neepawa, 5500HR, 5601HR, and 5602HR (Gilbert, unpublished data). This FHB resistance may come from Frontana, which is present in the pedigree of many of these wheats (Gilbert and Tekauz, 2000). This study examines the genetic basis of FHB resistance in the Canadian spring wheat variety Kenyon.

## Materials and methods

### Population

A F_9_-derived recombinant inbred line (RIL) population consisting of 125 lines from the cross Kenyon x 86ISMN 2137 was tested in this study. Each RIL was generated by single seed descent from a unique F2 individual. Kenyon is a Canada Western Red Spring (CWRS) variety with the pedigree Neepawa^*^5/Buck Manantial (Hughes and Hucl, 1991). Neepawa was the most widely grown CWRS variety in the 1970s to mid-1980s in western Canada (McCallum and DePauw, 2008), and a prominent in the pedigree of many current CWRS wheat varieties. Buck Manantial was the source of the leaf rust resistance gene *Lr16* in Kenyon. 86ISMN 2137 is a spring wheat line with resistance to tan spot (Singh and Hughes, 2005), Septoria nodorum blotch (Feng et al., 2004), and Septoria tritici blotch (McCartney, unpublished data), however, its pedigree is unknown.

### Phenotyping

The RIL population, parents, and checks were tested in six environments over 2012 and 2013 (Carman MB 2012, Winnipeg MB 2012, Carman MB 2013, Charlottetown PEI 2013, Ottawa ON 2013, and Winnipeg MB 2013). The check lines were 5602 HR, 93 FHB 37, AC Barrie, AC Cora, AC Morse, AC Vista, CDC Teal, ND 2710, Neepawa, Roblin, Shaw, and Snowbird. Entries were randomized in an alpha lattice with 12 incomplete blocks of 12 plots each, with 3 replicates per environment. The experimental unit was a 1 m row.

The date of 50% anthesis was recorded for each plot in all locations, except Charlottetown, PEI. On this date, anthesis had begun for 50% of the main tillers in that plot. Carman and Winnipeg tests were spray inoculated twice [on the recorded anthesis date and 2–3 days later (confirmed for Carman and Winnipeg)]. The inoculum consisted of the isolates M7-07-1 (3-ADON), M9-07-1 (3-ADON), M1-07-2 (15-ADON) and M3-07-2 (15-ADON) for the Carman nurseries and the Winnipeg 2012 nursery. In Winnipeg 2013, the isolates used were M9-07-01 (3-ADON) and M1-07-02 (15-ADON). The inoculum concentration was adjusted to 50,000 macroconidia per L and applied at a rate of 50 ml per row with a backpack sprayer. During the period the population was being inoculated, a misting system irrigated each day for 10 min every hour over a 12 h period (6:00 pm–6:00 am) in Carman. This misting system kept the plots wet overnight to promote *F. graminearum* infection. In Winnipeg, plots were irrigated with a sprinkler head irrigation system for 1 h following inoculation. The goal of this irrigation was to maintain wet soil to result in a humid micro-environment in the nursery, rather than to directly apply water to the canopy over the entire night.

In PEI, five *F. graminearum* isolates were used to produce macroconidia for inoculation. Inoculum was prepared to 50,000 macroconidia per ml and inoculated on plots three times per week over the course of flowering in the trial. Inoculum was applied with a standard pesticide sprayer delivering 200 L/ha water. The field was misted for 2 min bursts at a rate of 650–700 L/ha; misting was done every half hour from hour 7:00–10:30, at 15 min intervals thru to 19:00, half hour intervals to 21:00 and then on an hourly basis until 7:00. Misting nozzles were Naan Dan 501/2 (yellow) (Southern Drip Irrigation Ltd, Lethbridge, Alberta) spaced 10 feet apart in rows which ran down the center of the plots to ensure complete and overlapping coverage.

The Ottawa FHB nursery was inoculated by spreading infected corn and barley kernels on the soil surface as described by Xue et al. (2006). The corn and barley was inoculated with a mixture of three *F. graminearum* isolates (DAOM178148, DAOM232369, and DAOM212678; Canadian Collection of Fungal Cultures, AAFC, Ottawa, Canada), colonized, and then dried down. Inoculated corn is spread between the rows at a rate of 80 grams/meter^2^ at about 6 weeks after planting. Plots were irrigated twice daily for 30 min with irrigation sprinklers to promote conditions favorable for infection by *F. graminearum*.

Plant height, anthesis date, FHB incidence, and FHB severity data were collected. Plant height was measured from the soil surface to the top of main tiller spikes (excluding awns if present). FHB incidence was the percentage of spikes with visual FHB symptoms. FHB severity was the percentage of the spike with visual FHB symptoms, when only considering diseased spikes. FHB incidence and severity data were converted into FHB visual rating index (VRI). VRI = (FHB incidence ^*^ FHB severity)/100.

### Statistics

Least squares means were calculated for anthesis date, plant height, and VRI with JMP Genomics 6.0 (SAS Institute Inc.) using a mixed model. Wheat lines were considered fixed effects, and environment, rep, and incomplete block were considered random effects.

### Genotyping

Ninety-seven RILs were genotyped with a combination of microsatellite, diversity array technology (DArT) (Akbari et al., 2006), and single nucleotide polymorphism (SNP) markers. Genomic DNA was extracted from freeze-dried leaf tissue with the DNeasy 96 Plant Kit (Qiagen, Toronto, Canada). DNA was quantified with PicoGreen stain (Molecular Probes, Inc., Eugene, Oregon, USA). SNP markers were genotyped on the RIL population and parents using the Illumina Infinium 9K wheat SNP beadchip (Illumina, San Diego, CA) (Cavanagh et al., 2013). The raw data were analyzed with GenomeStudio V2011.1 software (Illumina, San Diego, CA). The genotype calls from GenomeStudio were converted into allele scores for linkage mapping in Excel. Markers with greater than 10% missing data or strong segregation distortion were excluded from mapping. Microsatellite markers were tested as previously described (McCartney et al., 2004).

### Linkage and QTL analysis

The linkage map was developed with MapDisto version 1.7.7 (Lorieux, 2012). Linkage groups were initially formed with stringent LOD and recombination fraction thresholds and gradually relaxed to a minimum LOD score of 4 and a maximum recombination fraction of 0.20 cM. Loci were ordered using a combination of the “AutoMap,” “Order sequence,” and “Compare all order” functions. The “Branch and Bound II” and “Seriation II” ordering methods were used in combination with the sum of adjacent recombination fractions (SARF) and count of crossover events (COUNT) as fitting criteria. For each linkage group, the shortest linkage map was selected from the linkage map solutions generated using these different mapping algorithms and criteria. The Kosambi mapping function was used to calculate map distances (cM) from recombination fractions.

QTL analysis was conducted with QTL IciMapping version 4.0.6.0 (Li et al., 2008) using interval mapping (IM) and inclusive composite interval mapping (ICIM). For linkage bins with more than one marker, a single marker was selected with the least missing data to represent the linkage bin for QTL analysis. Analysis for additive effect QTL was conducted with 0.1 cM steps and the 5% LOD significance threshold was calculated with 10,000 permutations. The LOD threshold for declaring QTL was 3.22 for additive effect QTL analysis for both IM and ICIM based upon this permutation analysis. Additive effect QTL were reported when the LOD score exceeded 3 in two or more environments, or one or more environments plus the pooled dataset, based upon IM or ICIM. For the reported QTL, QTL statistics were reported for environments in which the LOD score exceeded 2.

Analysis for epistatic QTL was conducted with 2.0 cM steps and a default LOD significance threshold of 5.0. Determining a 5% LOD significance threshold by permutation analysis was not possible for epistatic QTL analysis because of the computational power required. Linkage maps and QTL scans were illustrated with MapChart v. 2.2 (Voorrips, 2002). For anthesis date and plant height, epistatic QTL were reported when the LOD exceeded 3.5 in four or more environments, or three environments plus the pooled dataset, based upon IM or ICIM. For FHB resistance, epistatic QTL were reported when the LOD exceeded 3.5 in seven or more combinations of environment (individual environments or pooled dataset) by traits (VRI, FHB incidence, FHB severity). The more stringent criteria was applied to FHB resistance because the three FHB resistance traits were pooled for considering QTL. For the reported QTL, QTL statistics were reported for environments in which the LOD score exceeded 3.5.

### Cytology

To confirm the presence of a reciprocal translocation, immature spikes were harvested from F_1_ plants. Spikes were fixed (9 95% ethanol: 6 chloroform: 1 glacial acetic acid) at −20°C for 24 h and stored in 70% ethanol at −20°C, changing the ethanol once a day for 3–4 days. Anthers were macerated in acetocarmine to liberate pollen mother cells (PMCs) and stain the chromatin. PMC preparations were warmed on a hot plate and gently squashed to spread the chromosomes. Cells in metaphase I of meiosis were analyzed under a compound microscope to confirm the presence of quadrivalents.

## Results

### Phenotypic analysis

**Figure 1:**
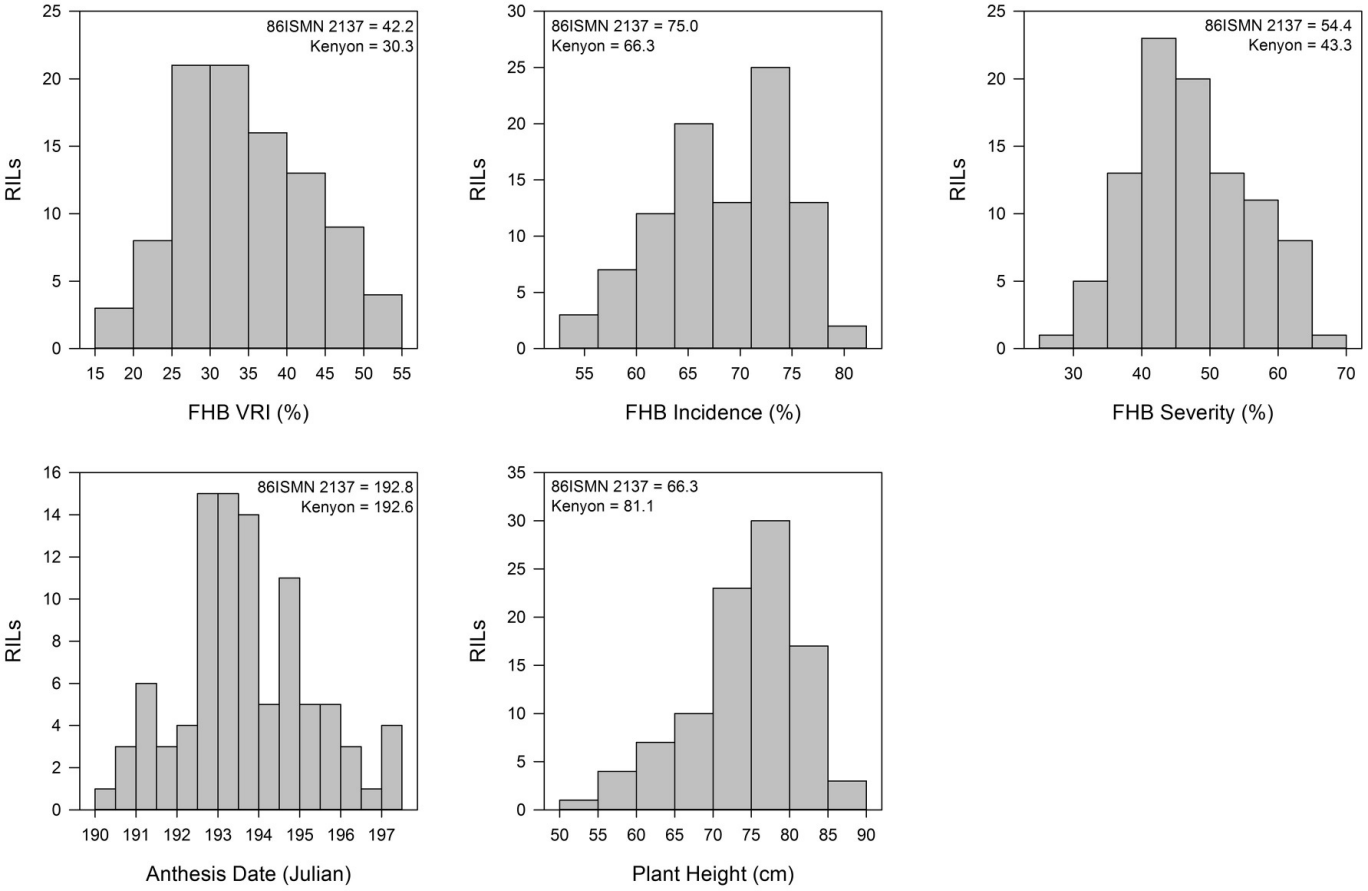
**Histograms of Fusarium head blight (FHB) Visual Rating Index (VRI) for the Kenyon/86ISMN 2137 RIL population in six environments over 2012 and 2013**. Means of the parents are indicated.

Trait data histograms for pooled datasets of the Kenyon/86ISMN 2137 RIL population are reported in Figure 1. Histograms for anthesis date, plant height, VRI, FHB incidence, and FHB severity from each FHB nursery are presented in Supplementary Figures S1–S5, respectively. All traits were approximately normally distributed. The earliest and latest RILs flowered within 8.3 days of each other (Figure 1, Supplementary Table S1). Kenyon and 86ISMN 2137 had very similar flowering dates, but some transgressive segregation was present in the population. Plant height varied widely with the shortest and tallest RILs differing by 36.5 cm (Figure 1, Supplementary Table S1). There was some transgressive segregation for plant height, but most RILs had means within the means of the parents.

The resistant checks 93FHB37 and ND 2710 had the lowest FHB VRI, incidence, and severity in the field FHB nurseries (Table 1, Supplementary Table S1). AC Vista, Roblin, and CDC Teal were the most susceptible to FHB based upon the VRI, incidence, and severity data, which was consistent with previous experience with these lines. The varieties 5602HR, AC Barrie, Neepawa, Snowbird, and AC Cora had intermediate FHB data. AC Morse, the sole durum wheat check, had intermediate to moderate susceptibility based upon VRI data, which is also typical. However, it should be noted that AC Morse would typically have very high Fusarium-damaged kernels (FDK) and deoxynivalenol (DON) accumulation relative to bread wheats with similar VRI scores. Cumulatively, these findings were all consistent with past experience with these wheat lines in FHB nurseries.

**Table 1:** **Least-squares means of checks and descriptive statistics of the Kenyon/86ISMN 2137 RIL population for FHB Visual Rating Index in field nurseries and pooled over environments**.

**VISUAL RATING INDEX (VRI)**							
	**Car12^a^**	**Wpg12**	**Car13**	**Ott13**	**PEI13**	**Wpg13**	**Pooled**
**POPULATION**							
Mean	54.3	15.3	50.6	23.5	33.0	27.2	34.0
Minimum	28.5	2.7	13.4	5.6	20.9	6.5	17.3
Maximum	85.8	45.1	93.8	67.7	48.4	65.8	53.4
**CHECKS**							
86ISMN 2137	55.2	16.5	57.1	50.1	34.1	38.2	42.2
Kenyon	59.4	11.5	36.8	13.3	34.9	25.0	30.3
Neepawa	42.1	8.5	19.2	5.2	24.5	21.0	19.9
5602HR	18.8	4.7	14.4	6.4	26.2	18.8	14.9
93FHB37	13.0	1.1	4.3	3.1	24.2	6.9	8.3
AC Barrie	31.2	5.8	22.3	3.6	27.3	14.0	17.5
AC Cora	55.1	7.9	37.3	7.2	25.0	17.0	25.0
AC Morse	60.0	20.2	47.8	19.9	25.1	26.1	33.0
AC Vista	91.1	14.3	90.4	59.2	40.8	32.2	54.5
CDC Teal	63.5	7.1	78.0	14.8	34.2	49.5	41.0
ND 2710	6.6	4.1	1.5	−4.0	22.7	5.5	5.8
Roblin	71.4	21.5	50.8	43.7	39.9	38.7	44.4
Shaw	61.2	18.5	47.2	16.5	30.9	30.6	34.6
Snowbird	42.5	19.9	20.8	11.9	28.7	21.5	23.6

a *Car, Carman, MB; Ot, Ottawa, ON; PEI, Charlottetown, PEI; Wpg, Winnipeg, MB; 12, 2012; 13, 2013*.

The wheat line 86ISMN 2137 was amongst the most susceptible to FHB based on VRI in these field nurseries (Table 1). Kenyon was more resistant to FHB than 86ISMN 2137 on average, and had a VRI score less than 86ISMN 2137 in four of the six field tests. Kenyon also had a higher VRI score than Neepawa in every field test, which was unexpected given its pedigree (Neepawa^*^5/Buck Manantial). The mean of Kenyon/86ISMN 2137 RIL population was similar to its parents, but there were individual RILs which were more resistant and susceptible to FHB. This result suggested transgressive segregation for FHB resistance in this population.

Correlation analysis revealed a consistent negative correlation between FHB VRI and plant height (*r* = −0.55) (Supplementary Table S2). Anthesis date and VRI were not highly correlated, and the correlation was not consistent (Supplementary Table S2). For instance, FHB VRI and anthesis date were negatively correlated in Winnipeg 2012, Ottawa 2013, and Winnipeg 2013, but positively correlated in Carman 2013 and completely uncorrelated in Carman 2012.

### Linkage map

The Kenyon/86ISMN 2137 linkage map was 2647 cM in length and consisted of 25 linkage groups and 3081 loci. Most of the wheat genome was covered by 22 of the linkage groups, with chromosome 1A consisting of two relatively large linkage groups of 82 and 29 cM (Supplementary Table S3). The three remaining linkage groups were small (7, 15, 13 cM) and were assigned to 1D (as linkage group 1D.1), 3D (as linkage group 3D.2), and 5D (as linkage group 5D.1) chromosomes based upon comparison to published maps (Cavanagh et al., 2013).

**Figure 2:**
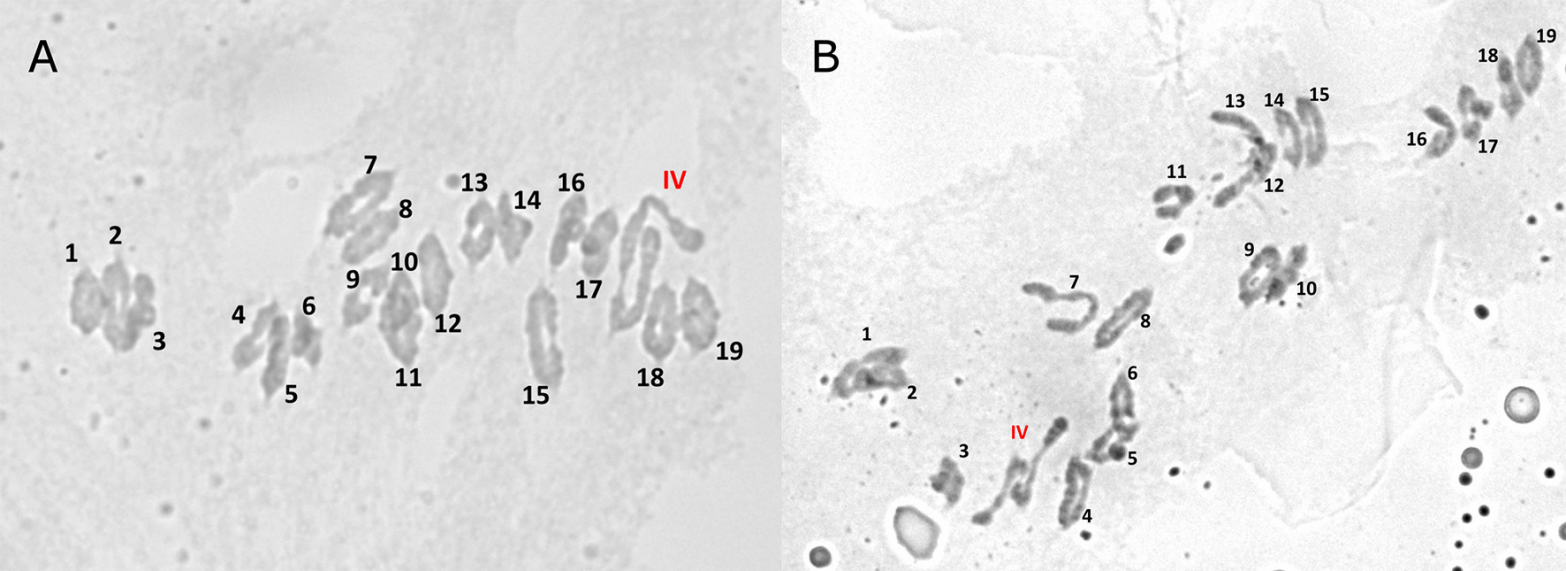
**Quadrivalents (labeled IV) observed during meiosis at metaphase I in pollen mother cell chromosome spreads (panels A and B) of F_1_ plants of the cross Kenyon/86ISMN 2137**.

A problem was identified in the 5B and 7B linkage maps. Under the linkage mapping conditions described above in the Materials and Methods, markers on chromosomes 5B and 7B formed a single linkage group. A reciprocal translocation was suspected in one of the parents. This was investigated by examining chromosome pairing during metaphase I in pollen mother cells. Cytology revealed the presence of quadrivalents during this phase of meiosis (Figure 2), which was also consistent with a translocation in one of parents. Markers on chromosomes 5B and 7B were subsequently separated with the maximum recombination fraction of 0.02 and a minimum LOD score of 5. This strategy successfully separated the markers for the two chromosomes, except for a single linkage group that represented the translocation breakpoint. The 5B and 7B markers, that were successfully separated based upon the stringent linkage group criteria above, were then mapped as outlined in the Materials and Methods. This resulted in four linkage groups that represented chromosomes 5B and 7B (i.e., a linkage group on either side of the translocation breakpoint for each chromosome). The markers in the translocation breakpoint linkage group were sorted into their appropriate chromosomes based upon the chromosome that these markers were previously mapped in other populations (Somers et al., 2004; Cavanagh et al., 2013). The linkage maps for chromosomes 5B and 7B were then re-calculated and a single linkage group was developed for each chromosome. The markers involved in the translocation breakpoint are indicated in Supplementary Table S3. These results were consistent with a reciprocal translocation in one of the parents of the mapping population, such that the 5B and 7B markers were genetically linked near the translocation breakpoint.

### Anthesis date

Three additive effect QTL for anthesis date were identified (Table 2). The QTL were located on chromosomes 2D, 4A, and 5B, and were named *QAnth.crc-2D, QAnth.crc-4A*, and *QAnth.crc-5B*. Kenyon alleles increased days to anthesis for *QAnth.crc-2D* and *QAnth.crc-5B*, and decreased days to anthesis for *QAnth.crc-4A*. *QAnth.crc-2D* and *QAnth.crc-5B* exceeded the LOD threshold more frequently than *QAnth.crc-4A*.

**Table 2:** **Additive effect QTL detected for plant height, anthesis date, FHB visual rating index, incidence, and severity in the Kenyon/86ISMN 2137 RIL population**.

		**IM^a^**	**IM**	**IM**	**IM**	**ICIM**	**ICIM**	**ICIM**	**ICIM**
**Trait–Dataset**	**Chr^b^**	**Pos^c^**	**LOD^d^**	**PVE^e^**	**Add^f^**	**Pos**	**LOD**	**PVE**	**Add**
**ANTHESIS DATE**									
Pooled	2D	36.4	5.97	25.14	0.80	36.5	10.08	27.46	0.84
Car12	2D	29.3	2.96	13.75	0.55	31.9	3.23	13.44	0.55
Wpg12	2D	36.4	5.07	21.86	1.16	34.8	10.75	21.12	1.14
Car13	2D	35.7	4.34	19.48	0.67	37.1	6.03	20.74	0.69
Ott13	2D	36.4	6.24	26.22	0.99	36.5	8.49	27.00	1.01
Wpg13	2D	36.4	4.69	20.41	0.66	36.8	6.88	24.56	0.72
Pooled	4A	103.6	2.69	13.22	−0.57	96.0	5.12	12.41	−0.56
Car12	4A					111.6	2.78	10.94	−0.49
Wpg12	4A	97.8	2.30	11.20	−0.82	98.0	4.15	7.15	−0.65
Car13	4A	102.9	2.17	10.58	−0.49	89.5	3.43	11.59	−0.52
Ott13	4A					92.9	2.71	7.85	−0.54
Wpg13	4A	101.8	2.66	12.14	−0.50	107.6	2.77	10.11	−0.46
Pooled	5B	72.0	3.73	16.50	0.65	72.0	5.60	13.50	0.59
Wpg12	5B	71.6	6.75	28.64	1.32	72.0	12.91	26.86	1.28
Car13	5B	73.2	2.61	11.97	0.52	73.3	2.78	8.71	0.45
Ott13	5B	72.2	3.61	16.18	0.78	72.1	4.31	12.22	0.68
Wpg13	5B					59.7	2.53	7.99	0.42
**PLANT HEIGHT**									
Pooled	1B	18.8	3.50	18.13	3.15				
Car13	1B	17.9	3.20	15.36	3.16	17.5	3.45	8.52	2.36
Ott13	1B	19.9	2.88	16.39	2.86				
Wpg13	1B	19.0	3.18	16.43	3.40				
Pooled	2D	27.0	5.14	25.31	3.73	26.7	7.73	26.42	3.81
Car13	2D	27.5	5.02	24.14	3.97	31.2	4.62	12.56	2.88
Ott13	2D	25.8	4.27	22.80	3.39	24.2	7.04	21.51	3.29
Wpg13	2D	27.1	4.55	22.41	3.97	26.2	6.52	24.54	4.16
Pooled	3D.1	62.5	3.08	15.62	2.95	54.7	4.67	13.64	2.76
Car13	3D.1	64.5	2.93	13.25	2.96	64.5	4.98	12.76	2.90
Ott13	3D.1	54.7	3.43	15.38	2.80	54.6	4.14	10.82	2.35
Wpg13	3D.1	63.1	2.87	14.15	3.18	54.7	3.79	12.13	2.95
Pooled	7B	38.0	2.20	10.12	2.35	38.0	4.51	13.04	2.67
Ott13	7B	38.0	2.38	10.91	2.33	39.1	4.30	11.28	2.37
Wpg13	7B					37.9	3.25	10.27	2.68
**FHB RESISTANCE**									
VRI_Pooled	2D	9.2	4.95	22.51	−3.97	8.5	4.16	13.69	−3.10
VRI_Car12	2D	8.9	2.97	14.01	−4.43	7.9	3.43	9.95	−3.73
VRI_Car13	2D	7.9	2.61	11.89	−5.77	7.9	2.04	8.30	−4.82
VRI_Wpg13	2D	7.9	3.96	17.49	−4.66				
SEV_Pooled	2D	8.7	4.98	22.60	−4.01	10.3	4.51	13.97	−3.15
SEV_Car12	2D	9.4	2.83	14.02	−3.60	7.9	2.30	7.67	−2.67
SEV_Car13	2D	7.9	2.81	12.77	−5.64	7.9	2.81	12.77	−5.64
SEV_Wpg13	2D	8.2	3.93	17.66	−4.32				
VRI_Wpg13	2D					17.5	3.41	10.31	−3.59
INC_Pooled	2D	24.5	4.81	24.05	−3.17	22.6	5.11	21.33	−2.98
INC_Car13	2D	24.3	2.70	14.20	−3.16				
INC_Ott13	2D	17.2	3.95	18.33	−4.42	18.3	5.26	20.81	−4.70
VRI_Pooled	2D					40.7	3.66	12.74	−3.02
VRI_Wpg12	2D	34.8	2.49	11.58	−3.13				
VRI_Ott13	2D	36.8	4.04	18.40	−4.97	36.8	4.04	18.40	−4.97
VRI_PEI13	2D					37.5	8.77	30.81	−3.42
VRI_Wpg13	2D					37.2	2.97	9.32	−3.43
INC_Car13	2D	40.0	2.17	11.39	−2.85				
INC_Ott13	2D	36.5	2.44	11.22	−3.47				
INC_PEI13	2D					37.7	7.21	25.47	−2.51
INC_Wpg13	2D	36.9	4.56	20.49	−4.16	36.9	6.20	25.53	−4.64
SEV_Pooled	2D	37.2	5.60	24.75	−4.23	41.6	4.74	15.13	−3.32
SEV_Ott13	2D	37.0	5.46	24.64	−7.01	37.0	5.34	17.84	−5.97
SEV_PEI13	2D	36.6	5.47	23.59	−3.24	36.0	9.71	31.75	−3.77
SEV_Wpg13	2D					37.0	4.98	18.71	−4.49
VRI_Car12	2D					108.3	4.16	14.78	−4.56
INC_Pooled	2D	88.7	2.59	12.28	−2.26				
INC_Car12	2D	110.7	3.84	21.86	−3.25	105.3	4.69	16.06	−2.79
SEV_Ott13	2D	77.8	2.58	12.48	−4.99				
VRI_Pooled	4A	51.1	3.27	14.65	3.21	53.6	3.12	9.54	2.59
VRI_Wpg12	4A	51.1	3.20	14.36	3.44				
VRI_Wpg13	4A	51.1	3.83	16.96	4.59	51.2	4.42	13.99	4.17
INC_Pooled	4A	51.2	2.80	12.92	2.32	51.1	2.54	9.43	1.98
INC_Car13	4A	51.1	2.30	10.55	2.73				
SEV_Pooled	4A	51.1	2.72	12.34	2.96	48.1	3.57	9.64	2.62
SEV_Wpg12	4A	51.2	2.38	11.10	3.86				
SEV_Wpg13	4A	51.1	3.92	17.33	4.28	51.2	3.44	12.46	3.63
VRI_Wpg12	4A					134.1	6.20	13.65	3.37
VRI_PEI13	4A	116.0	2.54	11.60	2.08	116.0	4.49	14.57	2.33
VRI_Wpg13	4A					137.1	2.29	6.30	2.81
INC_PEI13	4A	116.1	3.39	15.27	1.93	115.4	6.36	21.86	2.31
SEV_Wpg13	4A					136.0	2.10	6.70	2.67
VRI_Pooled	4D	11.6	2.97	13.41	3.11				
VRI_Car12	4D	11.6	2.66	12.09	4.17				
VRI_Wpg12	4D	11.6	3.11	13.99	3.44				
VRI_Wpg13	4D	20.5	2.04	9.44	3.45				
INC_Pooled	4D	11.6	2.82	12.76	2.34				
INC_Car12	4D	11.6	2.99	13.50	2.59	11.6	2.80	9.13	2.13
INC_Wpg12	4D	11.6	2.05	9.48	4.64	11.6	2.05	9.48	4.64
INC_Car13	4D	11.6	2.05	9.45	2.62				
INC_PEI13	4D	23.0	2.49	11.38	1.67				
SEV_Pooled	4D	11.6	2.48	11.33	2.88				
SEV_Wpg12	4D	11.6	2.41	11.01	3.90				
SEV_Ott13	4D					23.0	3.12	9.49	4.31
VRI_Ott13	5B	82.9	2.20	10.26	−3.68	88.3	2.42	9.95	−3.62
VRI_Wpg13	5B					94.9	2.07	6.48	−2.83
SEV_Wpg12	5B	110.0	2.71	12.36	−4.06	113.2	3.72	13.47	−4.24
SEV_Ott13	5B	82.1	3.06	14.46	−5.33	82.7	5.34	17.23	−5.81
SEV_Wpg13	5B					90.2	2.15	6.60	−2.65
VRI_Pooled	7D	74.5	4.15	18.23	−3.58				
VRI_Car12	7D	76.3	3.76	16.65	−4.86	76.9	3.94	11.48	−4.05
VRI_Wpg12	7D	76.9	3.14	14.10	−3.45	76.3	7.95	18.30	−3.91
VRI_Car13	7D	74.4	2.67	12.18	−5.85	74.4	2.67	12.18	−5.85
VRI_Wpg13	7D	74.4	2.87	13.02	−4.02				
INC_Pooled	7D	73.0	2.97	14.33	−2.44				
INC_Car13	7D	71.9	3.35	15.85	−3.34	71.9	4.45	18.37	−3.59
INC_Wpg13	7D	74.3	2.52	11.62	−3.10				
SEV_Pooled	7D	76.9	4.39	19.17	−3.73				
SEV_Car12	7D	76.3	4.03	17.76	−4.09	76.3	4.76	18.90	−4.22
SEV_Wpg12	7D	76.9	4.20	18.43	−5.02	76.9	5.34	20.10	−5.24
SEV_Car13	7D	74.5	2.24	10.31	−5.07				
SEV_Wpg13	7D	74.5	2.67	12.17	−3.59				

a *IM, interval mapping; ICIM, inclusive composite interval mapping*.

b *Chrom, chromosome*.

c *Pos, position on linkage group (cM)*.

d *LOD, peak LOD score; LOD threshold (IM), 3.22, LOD threshold (ICIM), 3.22*.

e *PVE, phenotypic variation explained (r^2^; %)*.

f *Add, additive effect of allele substitution. The units are those of the respective trait. A positive sign indicated that the ‘Kenyon’ allele increased the respective quantitative trait, and vice-versa*.

Analysis for epistatic QTL identified additional QTL for anthesis date. Three digenic epistatic interactions were identified (Supplementary Table S4). The most consistent identified epistatic interaction was between loci on chromosomes 5B and 5D. The *r*^2^ value and estimated additive^*^additive effect of the interaction was generally higher for IM than ICIM. Other epistatic interactions were identified between loci on chromosomes 3B and 3D, and 3D and 7D. None of the additive effect QTL for anthesis date were detected as an epistatic QTL, and vice-versa.

### Plant height

Four additive effect QTL for plant height were identified (Table 2). These QTL were located on chromosome 1B, 2D, 3D (linkage group 3D.1), and 7B, and were named *QHt.crc-1B, QHt.crc-2D, QHt.crc-3D*, and *QHt.crc-7B*. The Kenyon allele increased plant height for each of these QTL, which is consistent with Kenyon being 15 cm taller than 86ISMN 2137 in field tests. The *QHt.crc-1B, QHt.crc-2D*, and *QHt.crc-3D* were detected by IM (exceed LOD 3.22 in at least one environment), while all four height QTL were detected by ICIM. One epistatic QTL interaction was detected between loci on chromosomes 2B and 6B. This interaction was consistently identified by IM, but half of the time by ICIM.

The minor 7B height QTL was located within the translocation breakpoint. The peak of this QTL (38.0 cM) was located 1.9 cM from the most likely position of the translocation breakpoint (36.1 cM). Given this, it is strange that a height QTL was not detected on chromosome 5B at the translocation breakpoint (33.2 cM). Examination of the allele scores between markers at position 33.2 cM on chromosome 5B and position 38.0 cM on chromosome 7B revealed five allele score differences for these locations amongst the RILs. The relatively weak effect of this QTL and the number of allele score differences between these locations was apparently sufficient to prevent detection of the height QTL at the translocation breakpoint on chromosome 5B. Given these results, we recommend caution regarding the accuracy of the location of this QTL (i.e., the QTL could be located at the translocation breakpoint on chromosome 7B, or possibly 5B).

### FHB resistance

Nine additive effect QTL for FHB resistance were identified by QTL analysis using IM and ICIM with the additive effect module of QTL IciMapping (Table 2). These FHB resistance QTL were located on chromosomes 2D, 4A, 4D, 5B, and 7D, and were named *QFhb.crc-2D.1* (chromosome 2D at 8.5 cM), *QFhb.crc-2D.2* (chromosome 2D at 20.7 cM), *QFhb.crc-2D.3* (chromosome 2D at 37.5 cM), *QFhb.crc-2D.4* (chromosome 2D at 98.2 cM), *QFhb.crc-4A.1* (chromosome 4A at 51.1 cM), *QFhb.crc-4A.2* (chromosome 4A at 124.4 cM), *QFhb.crc-4D, QFhb.crc-5B*, and *QFhb.crc-7D*. Kenyon alleles decreased FHB symptoms for *QFhb.crc-2D.1, QFhb.crc-2D.2, QFhb.crc-2D.3, QFhb.crc-2D.4, QFhb.crc-5B*, and *QFhb.crc-7D*, and increased VRI for *QFhb.crc-4A.1, QFhb.crc-4A.2*, and *QFhb.crc-4D*. *QFhb.crc-2D.3* was the most consistently detected. *QFhb.crc-4D* and *QFhb.crc-5B* were the least significant FHB resistance QTL. The other FHB resistance QTL were reasonably consistent and detected by both IM and ICIM.

Additional QTL for FHB resistance were identified by digenic epistasis QTL analysis. Four digenic epistatic interactions were identified between loci on chromosomes 1A and 4B, 1B (near *QHt.crc-1B*) and 7B (*QHt.crc-7B*), 2B and 6B, and 2B and 6D (Supplementary Table S4). All four interactions were detected in approximately the same number of environments. None of the FHB resistance QTL detected by digenic epistasis loci were identified by additive effect QTL analysis. Similar to anthesis date, the *r*^2^ value and estimated additive^*^additive effect of the interactions was generally higher for IM than ICIM.

## Discussion

This study is the first study of native FHB resistance in western Canadian spring wheat. Nine FHB resistance QTL were detected in total. Kenyon contributed resistance at six of these QTL, which is consistent with Kenyon being more resistant than 86ISMN 2137 in these field FHB nurseries. QTL for FHB resistance were generally independent of QTL for anthesis date or plant height, except chromosome 2D. This is discussed in greater detail below. Kenyon contributed the resistance allele at *QFhb.crc-7D*, one of the most consistently detected QTL in this study. *QFhb.crc-7D* mapped to the same location as *Qfhb.sdsu-7D* (Eckard et al., 2015), where the resistant allele came from the wheat lines Wesley-*Fhb1*-BC56 and AL-107-6106. *QFhb.crc-7D* mapped to the same location as a minor FHB resistance QTL in the Wangshuibai/Alondra's population (Jia et al., 2005).

The anthesis date QTL *QAnth.crc-2D* is likely caused by photoperiod sensitivity gene *Ppd-D1*. *QAnth.crc-2D* mapped to ~37.1 cM on the 2D linkage group in the Kenyon/86ISMN 2137 RIL population, which is between *Xgwm261* and *Xgwm484* (*Xgwm261*−19.7 cM–*QAnth.crc-2D*−10.3 cM–*Xgwm484*). This is the expected location of *Ppd-D1* based upon the Cappelle-Desprez (Mara 2D) RIL population (*Xgwm261*−22.3 cM–*Ppd-D1*−12.4 cM–*Xgwm484*) (Gasperini et al., 2012). Likewise, the plant height QTL *QHt.crc-2D* was detected in all environments in which plant height was measured, and in the pooled dataset. *Rht8* was suspected to be responsible for *QHt.crc-2D*, which is known to map near *Xgwm261* (Korzun et al., 1998). 86SIMN 2137 carries the 192 bp allele of *Xgwm261*, which is associated with the *Rht8* reduced height allele (Korzun et al., 1998; Worland et al., 1998). *Xgwm261* mapped to position 17.4 cM on chromosome 2D in the Kenyon/86ISMN 2137 population, which places *QHt.crc-2D* approximately 9.5 cM proximal of distal of *Xgwm261*. *Rht8* maps 1.95 cM proximal of *Xgwm261* based upon fine mapping (Gasperini et al., 2012). The location of *QHt.crc-2D* in this population is most likely due to the combined effect of *Rht8* and *Ppd-D1*, since *Ppd-D1* is known to have a pleiotropic effect on plant height by shortening the life cycle (Worland and Law, 1986).

Three FHB resistance QTL (*QFhb.crc-2D.1, QFhb.crc-2D.2*, and *QFhb.crc-2D.3*) mapped to a relatively small region of chromosome arm 2DS. Kenyon contributed FHB resistance at all three loci, and carries a tall allele at *Rht8* and *Ppd-D1b* (daylength sensitive allele). Given the close proximity of these FHB resistance QTL, it is difficult to conclusively determine whether they are truly distinct based on the present data. *QFhb.crc-2D.2* mapped 1.3 cM distal of the expected location of *Rht8* (position 19.4 cM) based on upon the position of *Xgwm261*. *QFhb.crc-2D.3* mapped approximately 2.1 cM distal of *QAnth.crc-2D* (i.e., the location of *Ppd-D1*). Given these results, *QFhb.crc-2D.2* and *QFhb.crc-2D.3* are likely due to the pleiotropic effects of *Rht8* and *Ppd-D1*. If true, *QFhb.crc-2D.2* and *QFhb.crc-2D.3* would be distinct from each other. *QFhb.crc-2D.1* mapped about 8.9 cM distal of *Xgwm261*, or about 11 cM distal of *Rht8*. This suggests that *QFhb.crc-2D.1* may be a distinct FHB resistance QTL from *QFhb.crc-2D.2*. FHB resistance QTL has been previously detected near *Rht8* (Somers et al., 2003; Handa et al., 2008; Löffler et al., 2009). Further, genetic study is needed to clarify the number of QTL affecting FHB resistance on chromosome arm 2DS and to differentiate genetic linkage vs. pleiotropy between FHB resistance, plant height, and photoperiod response.

*QFhb.crc-2D.4* mapped to the same region of chromosome 2D as the FHB resistance QTL present in Wuhan-1 (Somers et al., 2003). Kenyon carries the FHB resistance allele for this QTL. This result was unexpected since Kenyon has no common ancestry with Wuhan-1. It should be noted that *QFhb.crc-2D.4* was not consistently identified in all field tests in this study. This contrasts with the Wuhan-1 2DL FHB resistance QTL, which was a strong QTL in past research (Somers et al., 2003; McCartney et al., 2007). Additional research is underway to study native FHB resistance in western Canadian germplasm, which will hopefully confirm the presence of *QFhb.crc-2D.4* in other Canadian germplasm. It would also be valuable to know whether the Wuhan-1 2DL QTL has a stronger effect that the Kenyon allele at *QFhb.crc-2D.4*.

The relationship between the QTL identified in this study and previously published QTL was explored through comparative mapping. This relied upon simple sequence repeat (SSR) loci common between the maps and the SSR consensus map by Somers et al. (2004). 86ISMN 2137 contributed three FHB resistance QTL (*QFhb.crc-4A.1, QFhb.crc-4A.2*, and *QFhb.crc-4D*). *QFhb.crc-4A.1* mapped to a similar location as an FHB resistance QTL derived from *T. macha* in a “Hobbit Sib” (*T. macha* 4A) single recombinant chromosome doubled haploid (DH) population (Steed et al., 2005). *QFhb.crc-4A.2* mapped to a similar location as an FHB resistance QTL from Arina (Paillard et al., 2004). *QFhb.crc-4D* mapped near a FHB resistance QTL from DH181 (pedigree: Sumai 3/HY368) and CS-SM3-7ADS (Chinese Spring Sumai 3 chromosome 7A disomic substitution line) (Ma et al., 2006). Unfortunately the origin of 86ISMN 2137 is not known, but DNA marker data suggests that this line is not closely related to Canadian spring wheats. It is unlikely that these three QTL from 86ISMN 2137 are present in Canadian spring wheats. Kenyon contributed the FHB resistance QTL *QFhb.crc-5B*, which is approximately the same map location as a FHB resistance QTL detected in Patterson (Bourdoncle and Ohm, 2003). Patterson is a soft red winter wheat from Purdue University, USA.

The variety Kenyon (pedigree: Neepawa^*^5/Buck Manantial) is a backcross derived line of the variety Neepawa, in which the leaf rust resistance gene *Lr16* was introgressed from Buck Manantial. *Lr16* was previously mapped to the short arm of chromosome 2B (McCartney et al., 2005). Interestingly, Neepawa was more resistant to FHB than Kenyon in all six environments tested. This suggested that the higher VRI score of Kenyon relative to Neepawa could be due to the *Lr16* introgression. However, no FHB resistance QTL was detected on chromosome 2B in this study, which indicates that the introgression carrying *Lr16* does not have a major effect on FHB resistance. This is fortunate since *Lr16* is a useful leaf rust resistance when pyramided with *Lr34* (Hiebert et al., 2010). Presumably, other portions of the genome must differ between Neepawa and Kenyon that are responsible for the difference in FHB resistance. It should be noted that *Lr16* has been confirmed to segregate in the Kenyon/86ISMN 2137 RIL population and was mapped to its expected location on chromosome arm 2BS (McCartney unpublished data).

Interestingly, the digenic epistatic interaction between loci on 1B and 7B for FHB resistance (Supplementary Table S4) corresponded to two additive effect QTL for plant height *QHt.crc-1B* and *QHt.crc-7B*. This result supports that this digenic epistasis interaction is valid and is not a statistical artifact. None of the other epistatic QTL were detected as additive effect QTL. This raises the question whether these epistatic QTL are real or statistical artifacts. Given the relatively small RIL population used in this study, it is quite possible that some of the detected epistatic interactions could be false. Additional research is needed to resolve this issue. None of the additive effect FHB resistance QTL were involved in epistatic interactions. This is likely good news for the deployment of these QTL in wheat breeding because this would suggest that each of these QTL appear to function independently of each other.

This study provides insight into the genetic basis of native FHB resistance in western Canada's CWRS marketing class, which is Canada's largest marketing class of wheat. A thorough knowledge of FHB resistance is needed to retain native FHB resistance from Canadian wheats and pyramid this with FHB resistance from Asian spring wheats, such as Sumai 3, Wangshuibai, and others. The FHB resistance QTL from Kenyon are likely to be valuable to wheat breeders in other growing regions, who would like to utilize FHB resistance QTL from wheats with excellent bread making properties.

## Author contributions

CM planned and organized the study. AB, GF, RM, BM, and JG collected Fusarium head blight data. CH conducted the cytology experiments examining chromosome pairing during meiosis. CM and CP conducted DNA marker analyses. CM developed the linkage map and conducted QTL analysis. All authors contributed to and approved the final manuscript.

### Conflict of interest statement

The authors declare that the research was conducted in the absence of any commercial or financial relationships that could be construed as a potential conflict of interest.
