# Supplementary figures and images for: Fusarium Head Blight Resistance QTL in the Spring Wheat Cross Kenyon/86ISMN 2137
Source: Front Microbiol. 2016 Oct 13;7:1542. doi: 10.3389/fmicb.2016.01542 (PMC5061752; doi:10.3389/fmicb.2016.01542)

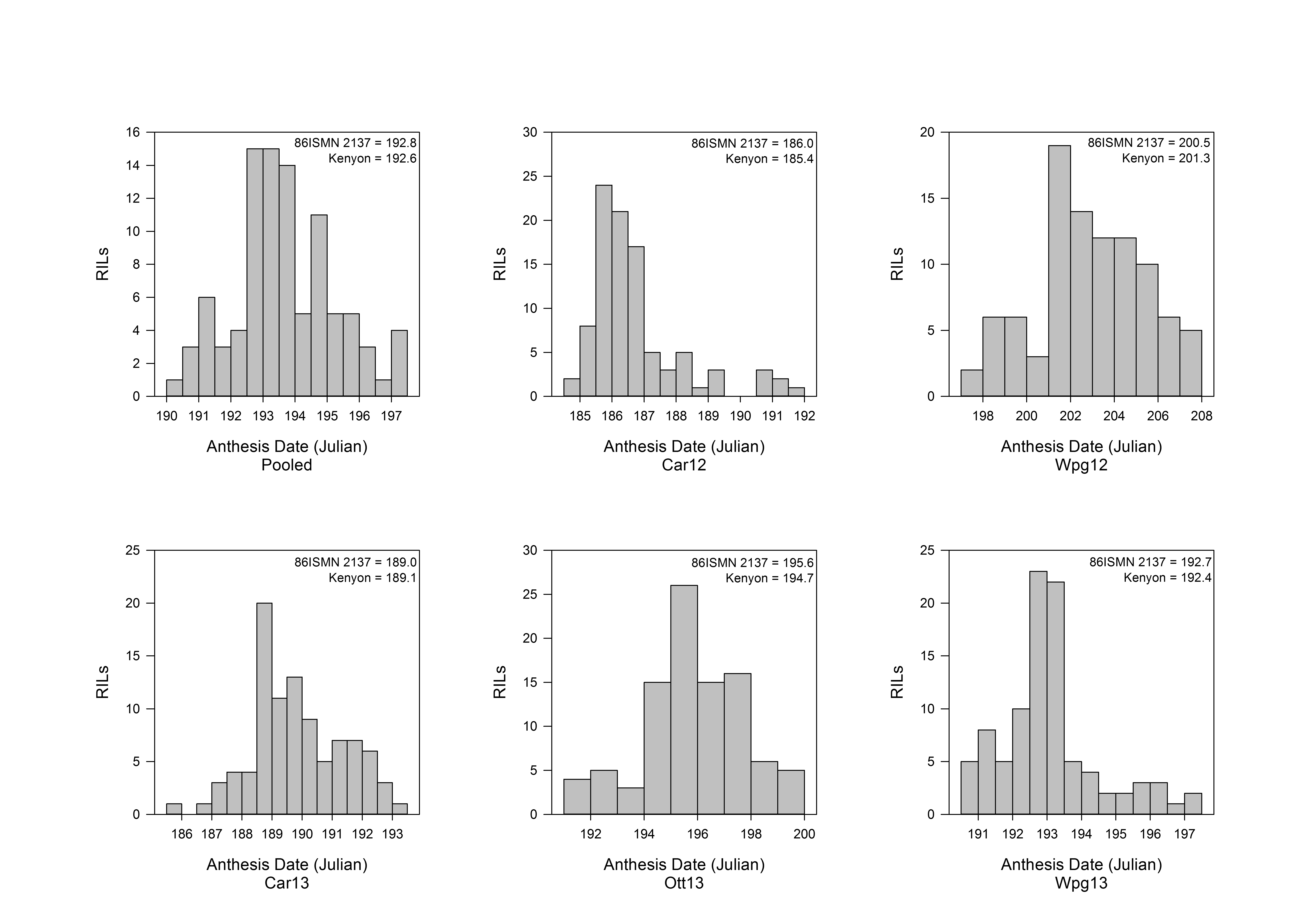

Supplement: Supplementary file 2 [file Image1.JPEG]

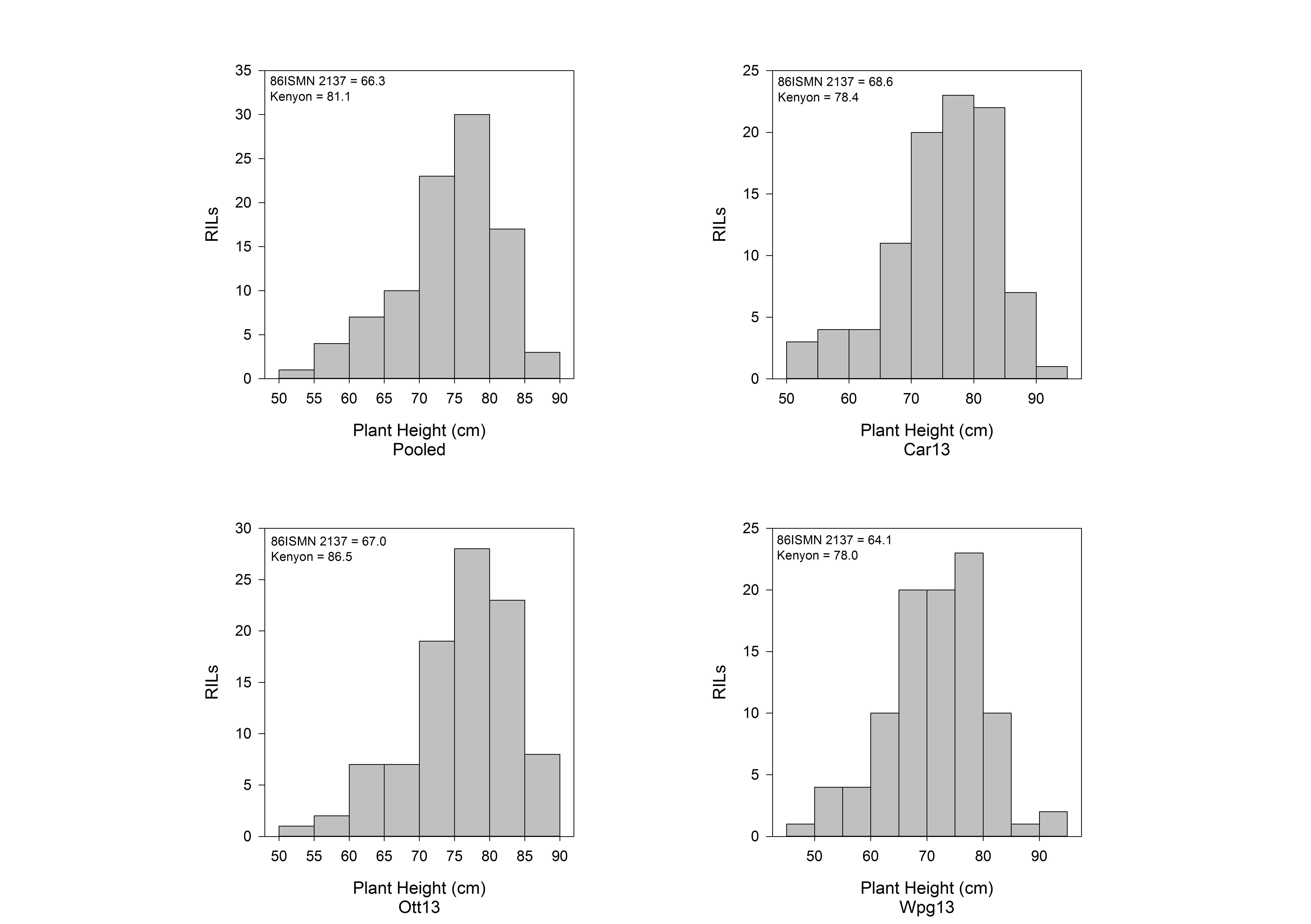

Supplement: Supplementary file 3 [file Image2.JPEG]

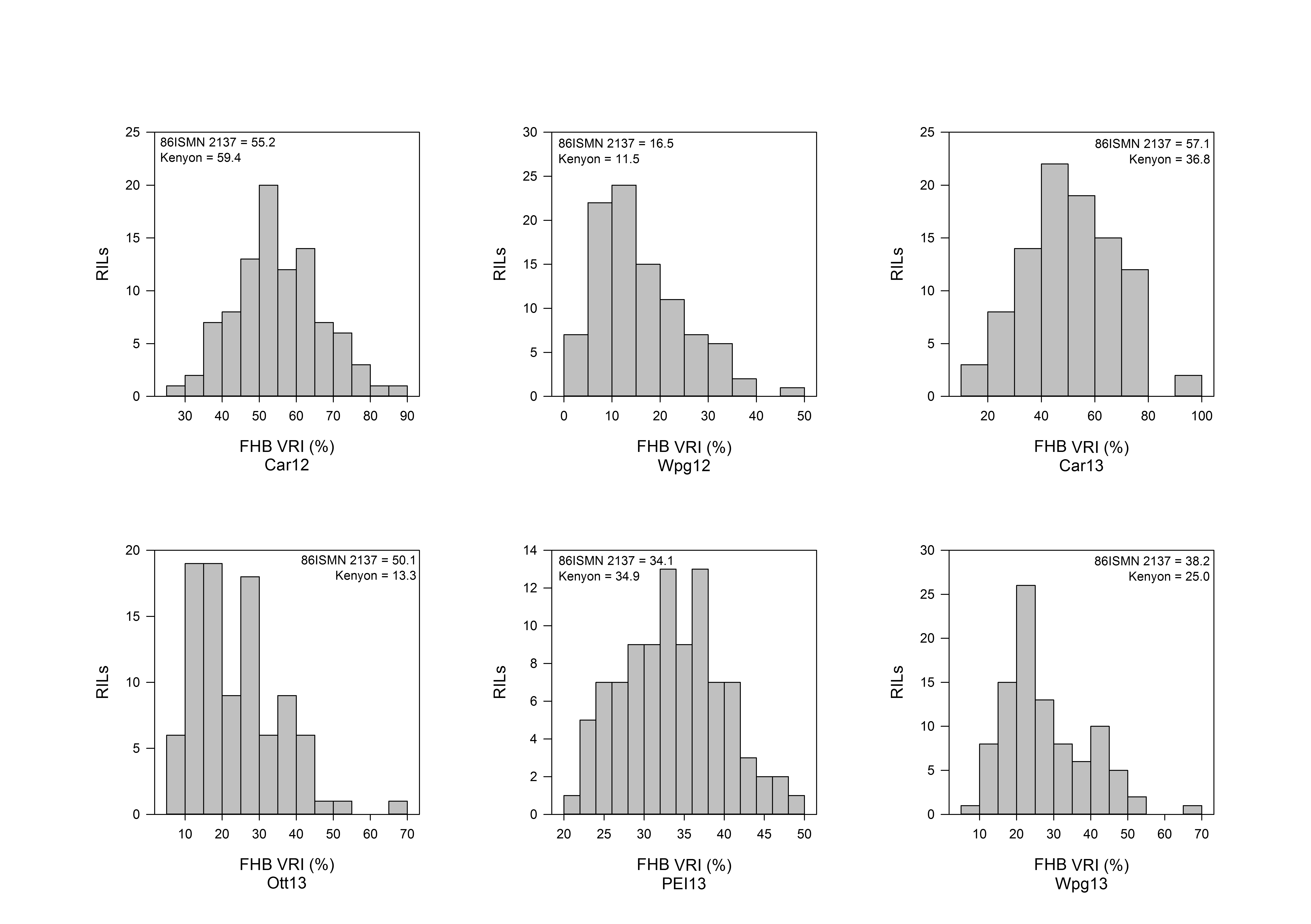

Supplement: Supplementary file 4 [file Image3.JPEG]

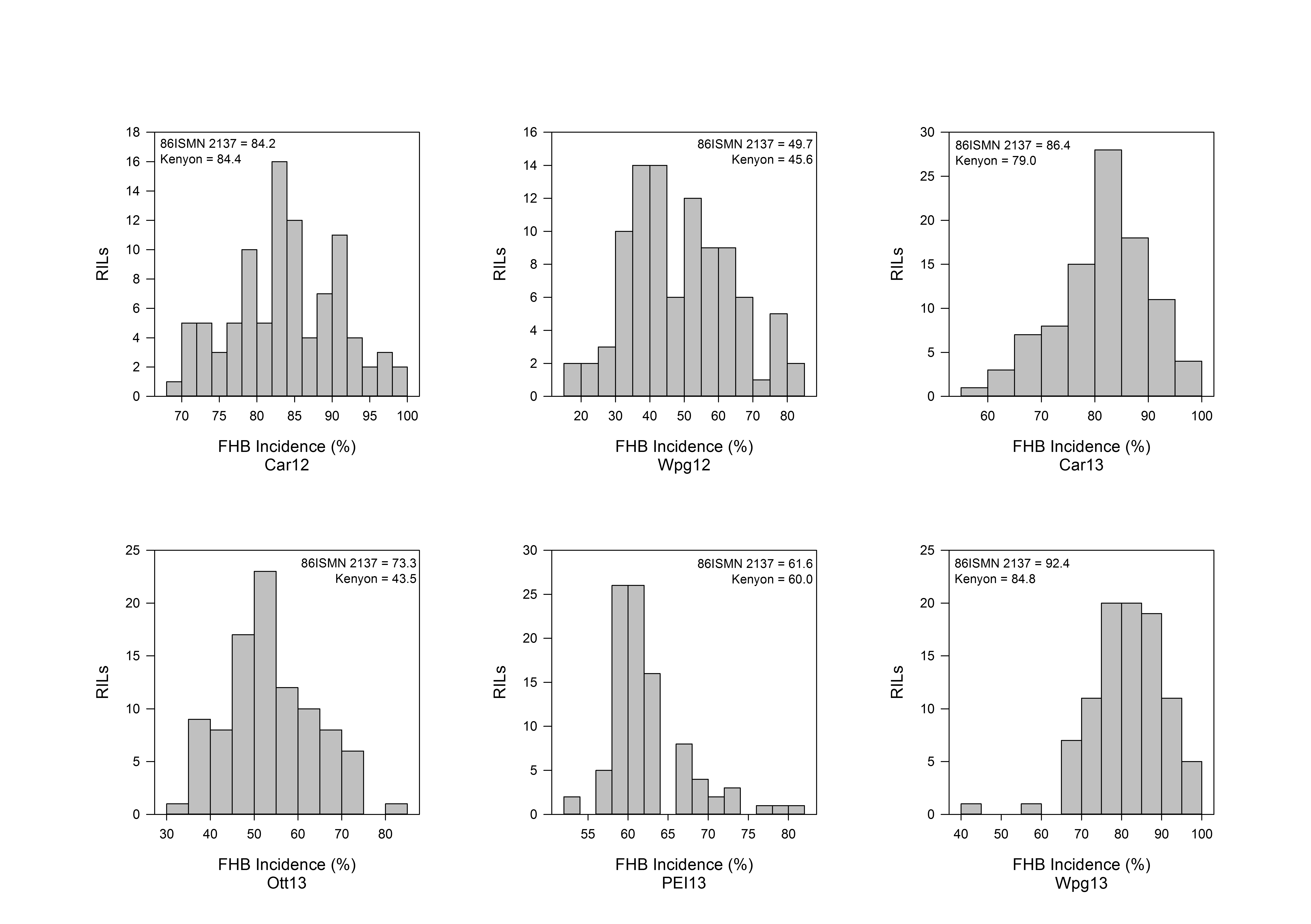

Supplement: Supplementary file 5 [file Image4.JPEG]

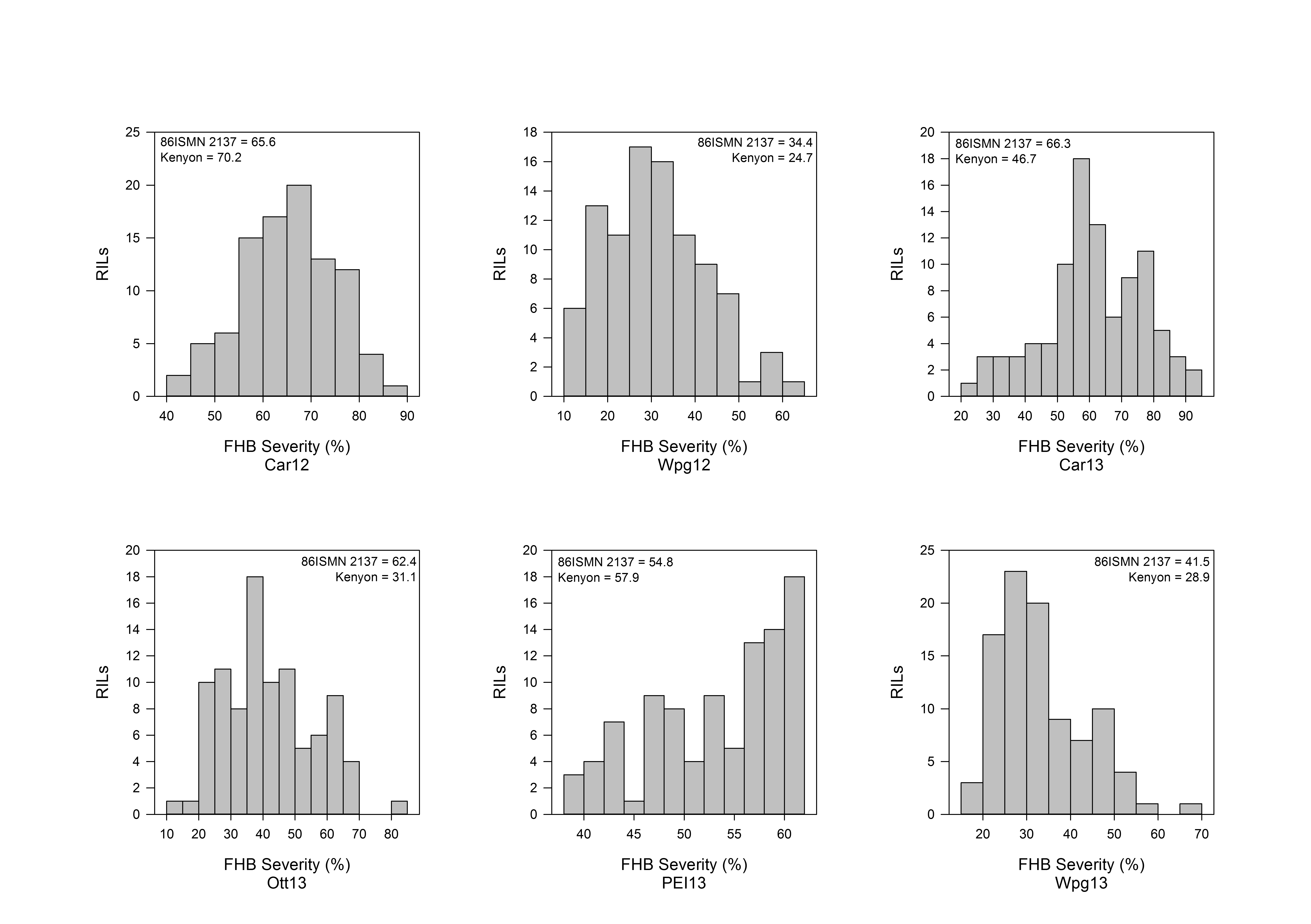

Supplement: Supplementary file 6 [file Image5.JPEG]
